# Complementary Feeding Practices in 80 Low- and Middle-Income Countries: Prevalence of and Socioeconomic Inequalities in Dietary Diversity, Meal Frequency, and Dietary Adequacy

**DOI:** 10.1093/jn/nxab088

**Published:** 2021-04-13

**Authors:** Giovanna Gatica-Domínguez, Paulo A R Neves, Aluísio J D Barros, Cesar G Victora

**Affiliations:** International Center for Equity in Health, Post-graduate Program in Epidemiology, Federal University of Pelotas, Pelotas, RS, Brazil; International Center for Equity in Health, Post-graduate Program in Epidemiology, Federal University of Pelotas, Pelotas, RS, Brazil; International Center for Equity in Health, Post-graduate Program in Epidemiology, Federal University of Pelotas, Pelotas, RS, Brazil; International Center for Equity in Health, Post-graduate Program in Epidemiology, Federal University of Pelotas, Pelotas, RS, Brazil

**Keywords:** complementary feeding, infant and young child feeding, socioeconomic factors, health equity, child nutrition

## Abstract

**Background:**

Adequate complementary feeding practices in early childhood contribute to better food preferences and health outcomes throughout the life course.

**Objectives:**

The aim of this study was to describe patterns and socioeconomic inequalities in complementary feeding practices among children aged 6–23 mo in 80 low- and middle-income countries.

**Methods:**

We analyzed national surveys carried out since 2010. Complementary feeding indicators for children aged 6–23 mo included minimum dietary diversity (MDD), minimum meal frequency (MMF), and minimum acceptable diet (MAD). Between- and within-country inequalities were documented using relative (wealth deciles), gross domestic product (GDP) per capita, and absolute (estimated household income) socioeconomic indicators. Statistical analyses included calculation of the slope index of inequality, Pearson correlation and linear regression, and scatter diagrams.

**Results:**

Only 21.3%, 56.2%, and 10.1% of the 80 countries showed prevalence levels >50% for MDD, MMF, and MAD, respectively. Western & Central Africa showed the lowest prevalence for all indicators, whereas the highest for MDD and MAD was Latin America & Caribbean, and for MMF it was East Asia & the Pacific. Log GDP per capita was positively associated with MDD (*R*^2^ = 48.5%), MMF (28.2%), and MAD (41.4%). Pro-rich within-country inequalities were observed in most countries for the 3 indicators; pro-poor inequalities were observed in 2 countries for MMF, and in none for the other 2 indicators. Breast milk was the only type of food with a pro-poor distribution, whereas animal-source foods (dairy products, flesh foods, and eggs) showed the most pronounced pro-rich inequality. Dietary diversity improved sharply when absolute annual household incomes exceeded ∼US$20,000. All 3 dietary indicators improved by age and no consistent differences were observed between boys and girls.

**Conclusions:**

Monitoring complementary feeding indicators across the world and implementing policies and programs to reduce wealth-related inequalities are essential to achieve optimal child nutrition.

## Introduction

During the first 2 y of life, all children must be optimally breastfed and receive an appropriate and diverse diet from 6 mo of age in order to achieve optimal growth and development (1–4). Departures from optimal growth vary in different groups of countries. In low- and middle-income countries (LMICs), stunting (low height-for-age) and micronutrient deficiencies are more prevalent owing to poor-quality diets (5).

The introduction of a healthy diet in early childhood contributes to better food preferences and health outcomes throughout the life course (5). In 2007, the WHO proposed a set of complementary feeding indicators for monitoring infant and young child feeding (IYCF) practices among children aged 6–23 mo (6, 7). The core indicators address the diversity [minimum dietary diversity (MDD)] and frequency [minimum meal frequency (MMF)] of child diets. A third indicator—minimum acceptable diet (MAD)—relates to child diets that met both diversity and frequency requirements. Analyses conducted in South Asia using national surveys found that children whose diets complied with the IYCF recommendations were less likely to be ill or malnourished (8, 9).

Socioeconomic inequalities represent a major threat to optimal feeding practices (10, 11). Using the 2007 definitions, a UNICEF report analyzed data from ≤87 national surveys in LMICs. The study found low overall prevalence levels for the indicators: 29.4% for MDD, 52.2% for MMF, and only 16% for MAD. The report also found that prevalence of the 3 indicators increased with household wealth within countries (12).

In 2018, UNICEF and the WHO updated the definitions of the 3 indicators, mainly in order to refine analyses of the diets of breastfed children (13, 14). A recent publication based on 49 national surveys from LMICs reported on MDD prevalence using the 2018 definition, but relied on the 2007 definition for measuring MMF, and MAD was calculated as the combination of these 2 indicators. The regions with the lowest and highest proportions of children aged 6–23 mo that met the 3 complementary feeding indicators’ requirements were Sub-Saharan Africa and Latin America & Caribbean, respectively. MDD prevalence ranged from 18% to 54%, MMF from 41% to 72%, and MAD from 9% to 40%. Stark disparities by wealth quintile were observed in most LMICs studied, particularly for MDD, which was also positively associated with Gross National Income (GNI) purchasing power parity (PPP) at country level (15). As far as we are aware, there are no multicountry analyses to date using the 2018 definitions of the 3 indicators.

The Sustainable Development Goals, part of the 2030 Agenda for Sustainable Development (16), call for action toward a better future for all, which includes appropriate diets for children, addressing goals 2 (zero hunger) and 3 (good health and well-being). Disaggregated analyses by socioeconomic indicators, including recent nationally representative surveys carried out in LMICs, are essential to track progress and identify challenges regarding complementary feeding practices. In the present analyses, we describe wealth-related inequalities in complementary feeding practices among children aged 6–23 mo in 80 LMICs. We used the 2018 definitions and provide breakdowns by wealth deciles, to allow greater granularity than wealth quintiles, and also by estimated absolute income of households in international dollars.

## Methods

The database of the International Center for Equity in Health (17) includes >400 national surveys with information on child health and nutrition in LMICs. We selected the most recent survey in each country, carried out since 2010, that included information on the 3 complementary feeding indicators described below, and sample sizes of ≥25 children aged 6–23 mo in each wealth decile. A total of 80 surveys were included, being 41 Demographic Health Surveys (DHSs) (18), 38 Multiple Indicator Cluster Surveys (MICSs) (19), and 1 modified version of the DHS from Ecuador (Encuesta Nacional de Salud y Nutrición 2012). DHSs and MICSs are highly comparable in terms of methodology and measurement protocols, allowing for the comparability of results (20). All surveys rely on multistage sampling procedures, selecting regions within countries, administrative units within each region (e.g., municipalities), census tracts within each administrative unit, and households within each tract. All women aged 15–49 y from selected households are invited for an interview on the nutrition and health of their under-5 children. Further information on survey methodology is available in each survey's published national reports.

### Complementary feeding indicators

Three complementary feeding indicators were estimated for children aged 6–23 mo, based on standardized questions about feeding practices during the 24 h preceding the survey (13). MDD was calculated as the proportion of children who consumed foods and beverages from ≥5 out of 8 food groups (see below). MMF was calculated as the number of breastfed children who consumed solid, semisolid, or soft foods at least twice (if aged 6–8 mo) or 3 times (if aged 9–23 mo), plus the number of nonbreastfed children who received ≥4 feeds, during the previous day (including ≥1 feed of solid, semisolid, or soft foods); the resulting sum is divided by the number of children aged 6–23 mo. Lastly, MAD was calculated as the percentage of children with satisfactory MDD and MMF, and who were either breastfed or had ≥2 non–human milk feeds in the previous 24 h.

For the 8 food groups used to calculate the MDD indicator, we also reported the percentage of children who during the previous day had consumed foods or beverages from each of the 8 food groups: *1*) breast milk; *2*) cereals and grains (grains, white/pale starchy roots, tubers, and plantains); *3*) legumes and nuts (beans, peas, lentils, nuts, and seeds); *4*) dairy products (milk, infant formula, yogurt, cheese); *5*) flesh foods (meat, fish, poultry, organ meats); *6*) eggs; *7*) vitamin A–rich fruits and vegetables; and *8*) other fruits and vegetables. Although 2 surveys (Papua New Guinea 2016 and Guyana 2014) did not collect data on yogurt, we decided to proceed with calculation of the MMF indicator because of the reportedly low frequency of yogurt consumption compared with other dairy products.

### Socioeconomic indicators and analyses

#### Wealth deciles

Household asset scores, generated through principal component analysis (PCA), were available in the DHS and MICS data sets. The PCA includes variables on household assets, building materials, and utilities like water and electricity, which are adjusted for the place of residence (21). The first component of the PCA, a continuous variable, was used to classify households into wealth deciles, with the first decile (D1) representing the poorest 10% of all families and the tenth decile (D10) representing the wealthiest 10% of all families.

#### Per capita gross domestic product

This indicator is expressed in current international dollars converted by PPP, and is the sum of the gross value added by all resident producers in the country plus any product taxes and minus any subsidies not included in the value of the products. The PPP conversion factor is a spatial price deflator and currency converter that eliminates the effects of the differences in price levels between countries (22). We obtained the gross domestic product (GDP) data through the wbopendata module 16.3, which draws from the core World Bank development indicators. It presents the most current and accurate global development data available, compiled from officially recognized international sources (23).

#### Absolute income for each wealth decile

This was calculated based on the national income levels obtained from the World Bank database (24), and national income inequality data collected from the standardized World Income Inequality Database (25). Dollar values (2011 PPP-adjusted international dollars) were then assigned to each household wealth decile, accounting for income's log-normal distribution (26).

#### Slope index of inequality

The slope index of inequality (SII) is a summary measure of absolute inequality, which is calculated through logistic regression models with the natural logarithms of the odds of the complementary feeding variables as the outcomes, and the wealth deciles as the independent variable. The SII represents the difference in the fitted value of the outcome between the highest and the lowest values of the wealth index scale (27), and is interpreted as percentage points (pp). SII values were pooled globally and regionally, and in the Supplementary Materials we also present pooled SII values by child age groups.

### Statistical analysis

For each country included in the analyses, we estimated the prevalence of the 3 indicators at the national level and according to sex and age (6–11, 12–17, and 18–23 mo) of the child. We also presented the diet indicators’ prevalence by wealth deciles and calculated the SII and its 95% CI. Next, we grouped the countries according to UNICEF world regions and World Bank income group classifications for the year of the survey (28, 29). Regional and income group estimates were weighted by the size of the population of children (i.e., aged 6–23 mo, boys and girls aged 6–23 mo, aged 6–11, 12–17, and 18–23 mo) in the year when the survey was conducted (30). Equiplot graphs were used to depict how weighted mean prevalence varied by wealth deciles.

We fitted polynomial equations for each complementary feeding indicator according to log GDP at national level, but this procedure did not improve the fit of the model compared with a linear equation. We present *R*^2^ values for the linear models for all children aged 6–23 mo and stratified by age group, which express the proportions of the variance of the complementary feeding indicators that are explained by GDP. Scatterplots were used to graph the associations. Fractional polynomials were used to describe nonlinear associations in graphical form of complementary feeding practices and absolute income. We also plotted within-country inequalities for the top 3 and bottom 3 countries in terms of each complementary feeding indicator's prevalence. Three countries (i.e., Papua New Guinea 2016, Sao Tome and Principe 2014, and Yemen 2013) had no available information on absolute income; hence, they were excluded only for the absolute income analysis. A logarithmic scale was used for the horizontal axes in the figures given the log-normal distribution of income. Lastly, the prevalence of consumption of each of the 8 food groups was calculated for the poorest and wealthiest deciles in each country and then grouped by world region.

All the analyses were performed in Stata 16.0 (StataCorp) considering the survey design, sampling weights, clustering, and stratification, and in RStudio version 3.6.0 (B Corps^TM^) for the absolute income graphs. The data used in our analyses are publicly available, and the institutions that conducted the surveys in each country handled the respective ethical clearance.

## Results

The most recent surveys were analyzed for 80 countries, with dates ranging from 2010 to 2019 (median: 2016). The number of children aged 6–23 mo in the national surveys ranged from 332 in Montenegro to 71,762 in India, with a median of 2581 children. **Supplemental Table 1** shows the surveys included in the analyses and sample sizes.

**FIGURE 1:**
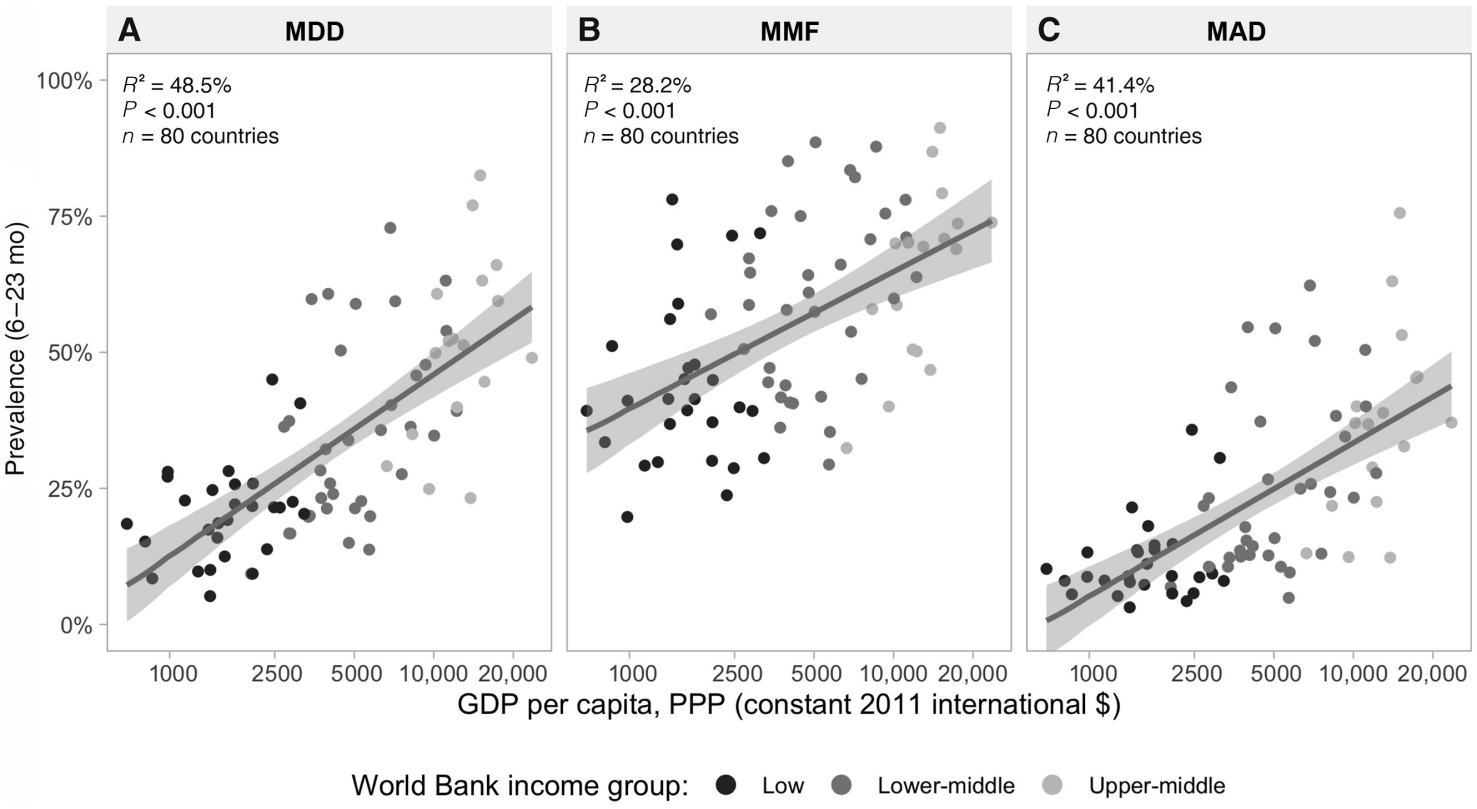
Country-level scatter diagrams of MDD (A), MMF (B), and MAD (C) according to per capita GDP (log scale) for 80 countries with available household surveys from 2010–2019 by World Bank income group. *R*^2^ values were derived from linear regression. GDP, gross domestic product; MDD, minimum dietary diversity; MMF, minimum meal frequency; MAD, minimum acceptable diet; PPP, purchasing power parity.

Our analyses included 90.3% of all low-income, 66.0% of all lower-middle-income, and 30.3% of all upper-middle-income countries in the world as of 2016. The numbers of countries with prevalence of ≥50% were 17 (21.3%) for MDD, 45 (56.2%) for MMF, and only 8 (10.1%) for MAD (**Supplemental Table 2**). **Figure 1** shows the ecological analyses with countries as the units. There were direct linear associations between log GDP per capita and MDD (*R*^2^ = 48.5%), MMF (*R*^2^ = 28.2%), and MAD (*R*^2^ = 41.4%). MDD was also correlated to MMF (*r* = 0.68; *P* < 0.001; data not shown). We also explored how the associations between GDP and dietary indicators were modified by age of the child (**Supplemental Table 3**). *R*^2^ values for MDD were equal to 41.3% for children aged 6–11 mo, 48.2% for those aged 12–17 mo, and 51.3% for those aged 18–23 mo. The corresponding *R*^2^ values were 23.5%, 26.1%, and 29.5% for MMF and 36.4%, 41.5%, and 42.0% for MAD, respectively. Therefore, associations between dietary indicators were stronger for older than for younger children. All *P* values were <0.001.

**Table 1** shows the prevalence of the 3 complementary feeding indicators by sex and age of the child according to world regions. In all regions, MMF showed higher prevalence than MDD, whereas the lowest prevalence was observed for MAD. Western & Central Africa was the region with the lowest mean of the weighted prevalence for the 3 indicators, followed by South Asia and by Eastern & Southern Africa. The highest mean prevalence for both MDD and MAD were observed in Latin America & Caribbean, and for MMF in East Asia & the Pacific, closely followed by Latin America & Caribbean. No consistent sex differences were observed in the analyses. At global level and for most regions, values of the 3 complementary feeding indicators, particularly MDD and MAD, increased between the first (6–11 mo) and second (12–17 mo) age groups with little change thereafter (*P* < 0.001).

**TABLE 1:** Weighted mean prevalence of complementary feeding indicators for all children aged 6–23 mo and stratified by sex and age group for 80 countries with available household surveys from 2010–2019, according to the regions of the world^1^

		All		Boys		Girls		6–11 mo		12–17 mo		18–23 mo	
World region	Countries, *n*	Mean	95% CI	Mean	95% CI	Mean	95% CI	Mean	95% CI	Mean	95% CI	Mean	95% CI
Minimum dietary diversity													
Western & Central Africa	21	19.0	16.6, 21.5	19.1	16.7, 21.6	19.0	16.5, 21.4	13.9	11.8, 16.1	22.4	19.3, 25.5	20.9	18.2, 23.5
Eastern & Southern Africa	18	24.0	19.9, 28.1	24.1	20.1, 28.0	24.1	19.8, 28.3	18.4	15.2, 21.6	28.6	24.4, 32.9	25.2	19.9, 30.5
Middle East & North Africa	6	36.2	26.5, 45.9	36.2	25.8, 46.7	36.3	27.4, 45.2	24.0	14.4, 33.5	40.9	30.6, 51.2	46.2	36.5, 55.8
Eastern Europe & Central Asia	10	50.3	36.5, 64.0	50.2	36.3, 64.2	50.5	36.6, 64.4	34.1	20.7, 47.6	59.5	44.4, 74.6	57.8	44.2, 71.3
South Asia	5	20.8	13.8, 27.7	20.5	13.1, 27.9	21.1	14.6, 27.5	11.0	5.8, 16.2	24.0	16.5, 31.4	27.9	19.5, 36.4
East Asia & the Pacific	10	50.6	42.2, 59.0	49.9	42.2, 57.6	51.4	42.1, 60.6	34.4	27.2, 41.6	57.6	48.5, 66.7	59.3	49.5, 69.2
Latin America & Caribbean	10	56.5	48.9, 64.1	53.5	46.1, 61.0	59.3	51.2, 67.4	38.9	33.9, 44.0	64.6	56.5, 72.8	67.2	56.3, 78.2
All regions	80	27.1	23.9, 30.3	26.8	23.7, 29.9	27.5	24.2, 30.8	17.8	15.4, 20.2	31.4	27.9, 34.9	32.6	28.8, 36.3
Minimum meal frequency													
Western & Central Africa	21	39.6	36.4, 42.7	39.0	35.8, 42.2	40.1	37.0, 43.3	41.6	38.1, 45.0	38.6	34.9, 42.3	38.4	34.6, 42.2
Eastern & Southern Africa	18	45.1	40.0, 50.3	44.9	39.8, 50.0	45.3	40.1, 50.5	47.6	42.2, 52.9	43.7	38.1, 49.3	44.3	38.3, 50.4
Middle East & North Africa	6	62.9	56.6, 69.3	63.3	56.8, 69.8	62.5	56.2, 68.8	58.3	51.2, 65.3	64.2	57.7, 70.7	67.1	61.7, 72.5
Eastern Europe & Central Asia	10	68.8	56.2, 81.5	68.1	55.7, 80.5	68.7	55.8, 81.7	58.7	46.0, 71.3	71.9	57.7, 86.1	74.7	62.8, 86.6
South Asia	5	43.2	28.1, 58.3	43.7	28.5, 58.9	42.6	27.6, 57.6	36.2	22.7, 49.7	43.8	27.4, 60.2	50.1	34.7, 65.4
East Asia & the Pacific	10	72.5	65.5, 79.5	73.1	66.2, 80.1	71.8	64.7, 79.0	70.4	64.3, 76.6	72.8	65.2, 80.4	74.0	66.4, 81.6
Latin America & Caribbean	10	71.5	63.9, 79.0	71.7	64.3, 79.2	71.2	63.5, 78.9	66.1	60.0, 72.2	72.5	64.4, 80.6	76.2	66.2, 86.2
All regions	80	48.7	45.2, 52.2	48.8	45.3, 52.3	48.5	45.1, 51.9	46.0	42.6, 49.4	48.7	45.0, 52.3	51.7	48.0, 55.4
Minimum acceptable diet													
Western & Central Africa	21	9.4	8.3, 10.6	9.0	7.9, 10.1	9.9	8.6, 11.1	7.9	6.6, 9.2	10.6	9.1, 12.2	9.7	8.5, 10.9
Eastern & Southern Africa	18	13.3	10.6, 16.0	13.5	10.6, 16.3	13.1	10.5, 15.7	11.5	9.3, 13.8	14.7	12.0, 17.4	13.7	10.0, 17.5
Middle East & North Africa	6	25.4	17.4, 33.5	25.6	16.9, 34.3	25.2	17.9, 32.6	19.6	11.6, 27.5	29.6	21.7, 37.5	28.1	19.3, 36.8
Eastern Europe & Central Asia	10	37.7	23.2, 52.2	37.2	22.7, 51.6	38.2	23.2, 53.3	27.3	14.1, 40.5	44.2	27.9, 60.5	41.8	26.6, 56.9
South Asia	5	12.1	5.1, 19.2	12.2	5.0, 19.4	12.1	5.1, 19.1	7.1	2.1, 12.1	13.2	5.4, 21.1	16.4	8.0, 24.8
East Asia & the Pacific	10	39.7	31.5, 48.0	39.6	31.7, 47.5	39.9	31.3, 48.5	28.0	20.8, 35.2	44.8	36.3, 53.4	45.8	36.4, 55.3
Latin America & Caribbean	10	43.7	36.4, 50.9	41.4	34.2, 48.5	45.7	38.0, 53.4	30.8	25.5, 36.2	48.9	41.1, 56.8	52.1	42.3, 61.8
All regions	80	17.3	14.4, 20.2	17.1	14.3, 19.9	17.5	14.6, 20.4	12.4	10.2, 14.6	19.3	16.1, 22.6	20.3	16.9, 23.7

^1^All estimates were weighted by the size of the population of children aged 6–23 mo, estimates by sex of the child were weighted according to the size of the populations of boys and girls aged 6–23 mo, and estimates by age group were weighted according to the size of the population of children in the respective age range.

**Table 2** shows results by wealth decile and region. The wealthiest children presented the highest mean values in all regions for the 3 indicators, and with a couple of minor exceptions the lowest values were observed in the poorest decile. All SII values were positive and significantly different from 0, indicating pro-rich inequality. MDD was more unequally distributed than MFF in 6 of the 7 world regions. The ranking of regions according to inequality varied by indicator, with Latin America & Caribbean being the most unequal region in terms of MMF, whereas MDD was most unequal in East Asia & the Pacific and in Eastern & Southern Africa. For MAD, 3 regions had similar levels of inequality of ∼10 pp (i.e., South Asia, Middle East & North Africa, and Western & Central Africa), whereas East Asia & the Pacific presented the highest inequality magnitude at 29.1 pp. **Supplemental Figure 1** presents these results in graphical form. Inequality in MDD was being mainly driven by the wealthiest groups in Sub-Saharan Africa and in Latin America & Caribbean, a pattern that has been described as “top inequality.” In contrast, in East Asia & the Pacific it was the poorest decile that had markedly lower prevalence than the rest of the population, a “bottom inequality” pattern. When countries were pooled according to World Bank groups, top inequality for MDD was particularly evident in low-income countries. Inequality patterns for MMF were not as evident (**Supplemental Figure 2**).

**TABLE 2:** Weighted mean prevalence of each complementary feeding indicator by wealth deciles and the SII for 80 countries with available household surveys from 2010–2019, according to world regions^1^

World region	Countries, *n*	Poorest	D2	D3	D4	D5	D6	D7	D8	D9	Wealthiest	SII	95% CI
Minimum dietary diversity													
Western & Central Africa	21	12.7	14.1	14.0	15.4	14.9	18.0	21.4	22.8	29.2	34.8	22.5	17.0, 27.9
Eastern & Southern Africa	18	14.2	16.4	17.6	19.9	21.5	24.2	26.4	29.7	36.5	45.1	30.2	24.3, 36.1
Middle East & North Africa	6	32.5	36.8	32.1	34.4	36.4	35.0	36.7	38.6	40.0	43.3	9.6	5.2, 13.9
Eastern Europe & Central Asia	10	41.7	44.0	48.7	47.8	52.6	53.1	51.2	54.9	58.5	52.6	14.6	8.2, 21.0
South Asia	5	13.9	16.2	17.7	19.0	20.0	21.9	23.8	25.1	25.8	27.7	14.8	13.3, 16.3
East Asia & the Pacific	10	30.7	44.1	45.0	47.6	48.9	55.0	53.3	56.8	61.8	64.2	30.4	22.6, 38.2
Latin America & Caribbean	10	46.2	57.5	49.9	60.6	53.8	59.7	60.8	57.6	58.4	72.6	17.4	5.4, 29.4
Minimum meal frequency													
Western & Central Africa	21	37.4	38.2	38.6	39.0	40.0	37.5	38.9	40.0	42.3	45.6	6.5	2.9, 10.1
Eastern & Southern Africa	18	38.8	40.6	41.5	42.5	44.3	48.0	43.9	49.2	52.9	56.2	17.4	13.8, 21.0
Middle East & North Africa	6	57.1	59.0	59.0	59.9	62.4	64.1	63.7	63.1	69.6	74.1	15.7	10.2, 21.3
Eastern Europe & Central Asia	10	66.9	62.3	67.7	67.6	68.7	70.5	71.1	72.5	72.2	71.6	8.9	4.6, 13.2
South Asia	5	36.8	39.4	41.3	40.8	43.3	43.2	44.9	46.4	49.0	51.2	14.2	12.3, 16.1
East Asia & the Pacific	10	67.0	67.1	66.7	73.1	72.0	74.3	74.2	72.1	78.8	79.3	13.6	10.0, 17.3
Latin America & Caribbean	10	62.0	66.7	64.6	71.7	73.1	77.8	72.7	82.0	78.7	79.9	20.5	16.1, 24.9
Minimum acceptable diet													
Western & Central Africa	21	6.4	7.5	7.1	7.8	7.0	8.3	10.3	11.1	14.0	17.7	10.8	7.3, 14.3
Eastern & Southern Africa	18	7.0	8.5	9.5	11.4	11.9	13.6	13.6	15.9	21.0	27.6	19.2	14.4, 23.9
Middle East & North Africa	6	20.4	26.1	22.0	24.8	25.3	24.5	24.6	27.8	29.1	33.8	10.2	5.0, 15.4
Eastern Europe & Central Asia	10	30.5	29.3	34.8	37.2	41.0	38.2	40.8	43.0	41.8	42.8	15.0	10.7, 19.2
South Asia	5	7.9	8.8	10.4	10.4	11.7	13.0	12.8	14.5	16.2	17.5	10.1	9.4, 10.9
East Asia & the Pacific	10	23.5	32.1	32.4	36.6	37.9	44.2	42.7	43.6	51.3	53.6	29.1	24.1, 34.2
Latin America & Caribbean	10	33.1	41.7	37.7	43.6	41.5	48.6	47.1	48.9	51.2	59.0	22.5	16.0, 28.9

^1^All estimates were weighted by the size of the population of children aged 6–23 mo. D, decile; SII, slope index of inequality.

**Supplemental Tables 4–6** and **Supplemental Figures 3–9** present more detailed results by country. Most countries showed pro-rich patterns for the 3 dietary indicators, and out of the 240 analyses performed only 2 countries presented significant pro-poor patterns (Kiribati and Guinea Bissau) for MMF.

To assess whether the age of the child modified the magnitude of dietary inequalities, we calculated the pooled value of the SII across all countries. The pooled SII values for all children were equal to 19.9 pp (95% CI: 8.2, 31.5 pp) for MDD, 13.8 pp (95% CI: 3.0, 24.7 pp) for MMF, and 16.6 pp (95% CI: 5.7, 27.6 pp) for MAD. After stratification by age group, the SII for MDD was equal to 16.4 pp for 6–11 mo, 21.9 pp for 12–17 mo, and 22.2 pp for 18–23 mo. The corresponding values for MMF were 13.2 pp, 14.4 pp, and 14.3 pp, and for MAD were 12.3 pp, 18.3 pp, and 19.8 pp, respectively (**Supplemental Table 7**).

The sharp inequalities in MDD led us to inspect the role of each of the 8 food groups included in this indicator. Breast milk was the only food with a pro-poor distribution. Inequalities in the consumption of cereal and grains, legumes and nuts, and vitamin A–rich fruits and vegetables were small, but for the other 4 food groups, particularly dairy products, they tended to be wide (**Table 3**). **Supplemental Table 8** presents additional results of food groups by world regions.

**TABLE 3:** Weighted mean prevalence of consumption of food groups in the poorest and wealthiest deciles, and SII, for 80 countries with available household surveys from 2010–2019^1^

Food group	Poorest decile, %	Wealthiest decile, %	SII (pp)	95% CI
Breast milk	36.1	28.6	−8.0	−9.4, −6.6
Cereal and grains	33.2	33.4	0.5	−0.1, 1.0
Legumes and nuts	8.6	9.4	0.9	0.1, 1.7
Dairy products	17.1	30.3	13.7	11.6, 15.7
Flesh foods	10.7	17.2	6.6	5.9, 7.3
Eggs	6.9	12.8	5.8	5.1, 6.6
Vitamin A–rich fruits and vegetables	18.5	20.2	1.2	0.5, 1.9
Other fruits and vegetables	8.7	15.1	6.4	5.6, 7.2

^1^pp, percentage points; SII, slope index of inequality.

**FIGURE 2:**
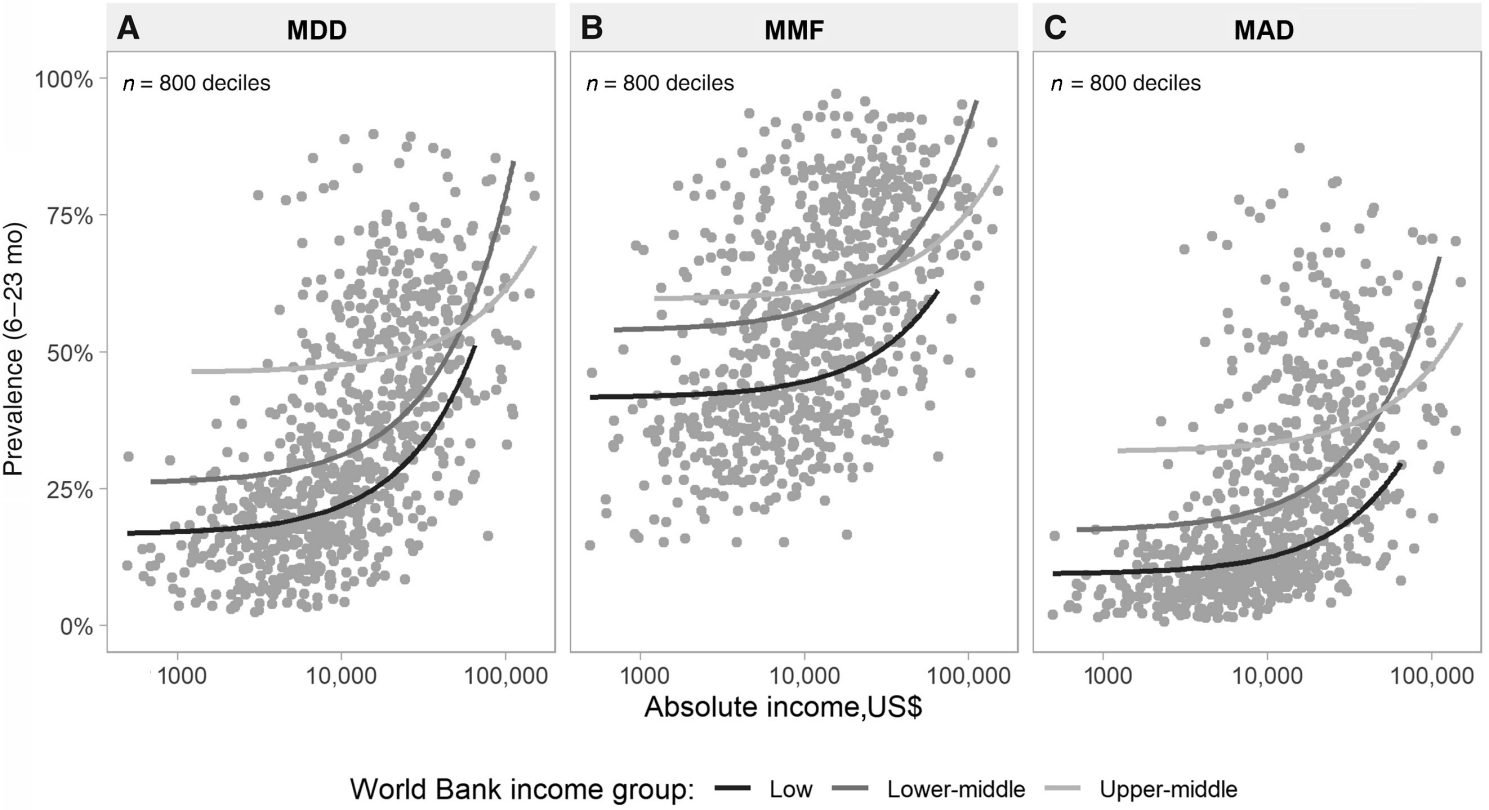
Absolute income (log scale) and complementary feeding indicators: MDD (A), MMF (B), and MAD (C), by World Bank income group. Each dot represents 1 wealth decile within the 80 countries with available household surveys from 2010–2019; 10 deciles/country. MDD, minimum dietary diversity; MMF, minimum meal frequency; MAD, minimum acceptable diet.

In the last set of analyses, the 3 complementary feeding indicators were plotted against absolute income. In **Figure 2**, the points represent the 800 deciles in all countries included in the analyses, and the lines are fractional polynomials for the 3 World Bank country income groups. For the same level of absolute household income, complementary feeding indicators tended to be highest in upper-middle-income countries, intermediate in lower-middle-income countries, and lowest in low-income countries, although at the upper end of the scale the patterns were similar in all middle-income countries. These results suggest that country characteristics may be driving complementary feeding patterns, beyond household income levels.

**FIGURE 3:**
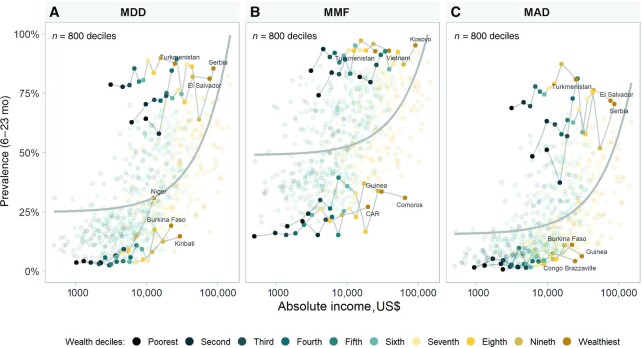
Absolute income (log scale) and the 3 top and bottom countries according to prevalence of MDD (A), MMF (B), and MAD (C), by World Bank income group. Each dot represents 1 wealth decile within the 80 countries with available household surveys from 2010–2019; 10 deciles/country. MDD, minimum dietary diversity; MMF, minimum meal frequency; MAD, minimum acceptable diet.

To further analyze these patterns, **Figure 3** was derived from Figure 2 to show the top and bottom 3 countries according to national prevalence of each complementary feeding indicator. Deciles with similar levels of household income showed much higher prevalence of dietary indicators in well-performing countries than in countries with poor performance. In the latter, even relatively wealthy households showed poor child diets. The most marked differences were observed for MDD.

## Discussion

Our analyses add to the literature by presenting, to our knowledge, the first report on the 3 internationally recommended indicators of complementary feeding for young children, using the 2018 definitions. The main difference between the previous definitions from 2007 and the current definitions refers to how breastfeeding was counted in terms of dietary diversity, as well as regarding how solid and semisolid foods were counted regarding meal frequency in nonbreastfed children. As a consequence of changes in these 2 indicators, the acceptable diet variable also changed.

Our pooled results showed that, across the 80 countries studied, only 1 in 4 children had diets that were sufficiently diverse, and 1 in 2 consumed the recommended number of meals per day. Regarding the age of the children, all 3 dietary indicators improved between the age groups of 6–11 mo and 12–17 mo, but remained stable—still at low levels—from 12–17 to 18–23 mo.

Being a combination of diversity and frequency, MADs were available to only 1 in 6 children. Earlier multicountry analyses using the 2007 indicators (12) or a combination of 2007 and 2018 indicators (15) had also shown that MDD had lower prevalence than MMF.

We found that East Asia & the Pacific was the region with the highest mean MMF, whereas the Latin America & Caribbean region had the best performance for MDD and MAD. In contrast, the earlier analyses by White et al. (12) showed that East Asia & the Pacific had the highest values for the 3 indicators. These differences may be due to changes in the definition of the indicators, and to the fact that the number of countries in the analyses varied between the 2 studies. In both studies the lowest means of the weighted prevalence for all 3 complementary feeding indicators were observed in the 2 Sub-Saharan African regions and in South Asia.

There were striking wealth-related inequalities, with pro-rich patterns present in between-country and within-country analyses, in all 7 world regions. Dietary diversity started to improve when absolute household income exceeded ∼US$20,000. The analyses by Baye and Kennedy (15) also found a direct association between GDP and diversity. Also, as the child's age increased, GDP was more strongly correlated with diet, especially with diversity.

Similar patterns were observed for within-country inequalities, which tended to be wider for diversity than for frequency in 5 of the 7 regions of the world, the exceptions being Latin America & Caribbean and Middle East & North Africa. In contrast to earlier analyses relying on wealth quintiles, our results by wealth decile were able to document socioeconomic gradients with greater granularity, while also confirming earlier reports of wider inequalities for dietary diversity than for frequency at global and regional levels (12, 15). Our observed patterns by sex and age group were in line with those presented by White et al. (12).

When we analyzed each of the 8 food groups, the widest inequalities were observed for consumption of animal-source foods, mainly caused by dairy product consumption, followed by flesh foods and eggs, and for consumption of fruits and vegetables other than those rich in vitamin A. Such inequalities were likely due to the high cost of these foodstuffs. Intake of animal-source foods, including dairy products, is fundamental because these constitute the richest sources of iron, zinc, calcium, vitamin A, and iodine, as well as high-quality protein. The consumption of animal-source foods has been associated with improved child growth, as well as cognitive and motor development in low-income countries (31, 32). There were virtually no socioeconomic differences in consumption of cereals and grains, legumes and nuts, and vitamin A–rich fruits and vegetables. Overall, cereals and grains tend to be the most affordable foodstuffs for practically all families, whereas the consumption of vitamin A-rich fruits and vegetables, and legumes and nuts might be low by children at this age, so that marked socioeconomic differences were not preset. The only food group with higher consumption among children from poor families was breast milk, a finding that is in accordance with the literature (33).

Our analyses focused on socioeconomic determinants of dietary adequacy. However, factors other than poverty affect the variability in complementary feeding practices across regions. Long-held cultural beliefs and stigmas are important drivers of the age at which some foodstuffs are introduced to young children (34). This may partly explain why younger children presented lower values for dietary diversity. Other factors affecting children's diets include lack of knowledge on the nutritional value of foods, inadequate child care, and limited availability of potentially important but neglected foodstuffs (2, 35).

The limitations of our study include the fact that indicators were derived from standardized questions on feeding practices during the 24 h preceding the survey applied to the survey respondents rather than actual observation and measurement of feeding patterns. Although it is possible that respondents may have under- or overreported the types of foodstuffs and frequencies of feeds, it should be noted that the prevalence of adequate complementary feeding was low, suggesting that poor diets are a major problem, particularly in low-income countries. Another limitation is that no data were available for 60 of all 140 LMICs (29); these included several upper-middle-income countries such as China and Brazil, where standardized surveys have not been conducted in recent years.

Among the strengths of our analyses, this is the first comprehensive study that we know of to have complied with the new definitions of complementary feeding indicators in LMICs, while also addressing both relative and absolute inequalities using different socioeconomic indicators and with greater granularity—wealth deciles rather than quintiles—than previously reported studies. In addition, we explored socioeconomic inequalities according to food groups.

Inadequate complementary feeding practices are major determinants of malnutrition, development, and mortality (1–4). At country level, targeted health and nutrition interventions delivered through large-scale programs, along with multisectoral programs promoting economic development, are essential to address the inequalities made evident in our analyses (36). Furthermore, these interventions and programs should consider the prevention of all types of malnutrition, be culturally sensitive, and be sustainable with local resources (37). Regular monitoring of dietary adequacy is an essential component for tracking progress toward the health and nutrition–related Sustainable Development Goals, particularly in order to counteract the huge impact of the COVID-19 pandemic on child nutrition (38, 39).

## Supplementary Material

nxab088_Supplemental_File

## Notes

Supported by Bill & Melinda Gates Foundation [Coverage & Equity of Reproductive, Maternal, Newborn, Child and Adolescent Health (RMNCAH) Outcomes: Research & Analysis from Pelotas] grant OPP1199234, Wellcome Trust grant 101815/Z/13/Z, and Associação Brasileira de Saúde Coletiva (ABRASCO).

Author disclosures: The authors report no conflicts of interest.

Supplemental Tables 1–8 and Supplemental Figures 1–9 are available from the “Supplementary data” link in the online posting of the article and from the same link in the online table of contents at https://academic.oup.com/jn.

Abbreviations used: DHS, Demographic and Health Survey; GDP, gross domestic product; IYCF, infant and young child feeding; LMIC, low- and middle-income country; MAD, minimum acceptable diet; MDD, minimum dietary diversity; MICS, Multiple Indicator Cluster Survey; MMF, minimum meal frequency; PCA, principal component analysis; pp, percentage points; PPP, purchasing power parity.
